# DNA damage repair machinery and HIV escape from innate immune sensing

**DOI:** 10.3389/fmicb.2014.00176

**Published:** 2014-04-22

**Authors:** Christelle Brégnard, Monsef Benkirane, Nadine Laguette

**Affiliations:** Laboratoire de Virologie Moléculaire, Institut de Génétique HumaineCNRS UPR1142, Montpellier, France

**Keywords:** DNA damage response, innate immunity, HIV, Vpr, G2/M arrest, SLX4 complex

## Abstract

Viruses have been long known to perturb cell cycle regulators and key players of the DNA damage response to benefit their life cycles. In the case of the human immunodeficiency virus (HIV), the viral auxiliary protein Vpr activates the structure-specific endonuclease SLX4 complex to promote escape from innate immune sensing and, as a side effect, induces replication stress in cycling cells and subsequent cell cycle arrest at the G2/M transition. This novel pathway subverted by HIV to prevent accumulation of viral reverse transcription by-products adds up to facilitating effects of major cellular exonucleases that degrade pathological DNA species. Within this review we discuss the impact of this finding on our understanding of the interplay between HIV replication and nucleic acid metabolism and its implications for cancer-related chronic inflammation.

## INTRODUCTION

Efficient human immunodeficiency virus (HIV) replication in target cells relies on its ability to use cellular resources and to overthrow host defense mechanisms. Indeed, viral fitness is defined by both the availability of cellular dependency factors and the ability to escape cellular blocks. One of the most challenging steps of HIV life cycle is the delivery of its single stranded RNA (ssRNA) genome and its conversion into double stranded DNA (dsDNA) in the host cell without inducing innate immune responses. Indeed, cellular “sensors” specialized in the recognition of foreign or pathological nucleic acids are present in different compartments through which viruses enter target cell. These nucleic acid sensors belong to the pattern recognition receptors (PRRs) family that recognize pathogen associated molecular patterns (PAMPs) and subsequently trigger a signaling cascade that culminates in the production of pro-inflammatory cytokines, including antiviral interferon (IFN; for review Kawai and Akira, 2006). Once recognition established, a signaling cascade is triggered, endowing an antiviral state and a cellular response aiming to clear the infection. In the course of reverse transcription of the HIV ssRNA genome into dsDNA, several intermediate, and sometime abortive, nucleic acid species are generated, including DNA:RNA hybrids, DNA flap structures and dsDNA. Exposure of these in the cytoplasm engages various sensors. Those include Toll like receptor 7 (TLR7) that detects viral RNA in endosomes (Beignon et al., 2005), Gamma-interferon-inducible protein 16 (IFI16) that recognizes virus-derived DNA in the cytoplasm of lymphoid quiescent CD4 T cells (Monroe et al., 2013) and the cyclic guanosine monophosphate-adenosine monophosphate synthase (cGAS; Gao et al., 2013).

To avoid recognition of reverse-transcription intermediates, viral genomes are protected within the capsid core. If uncoating is correctly orchestrated, this ensures delivery of fully reverse-transcribed viral genomes into the nucleus that are subsequently integrated into the host cell genome, a pre-requisite for the establishment of productive infection. However, as reviewed in Yan and Hasan within this issue, while few viruses efficiently undergo these steps, abortive infection events also occur, leading to accumulation of “junk” nucleic acid species in the host cell cytoplasm that may be detected and thereby have adverse effects on the infection. The frailty of viral reverse-transcription is highlighted by the plethora of antiviral factors that target this specific step. Those include three of the prototypical restriction factors. Although their mechanism of action being beyond the scope of this review, it is worthy to mention that Tripartite Motif 5 alpha (TRIM5α) causes untimely uncoating, leading to premature exposure of virus-derived nucleic acid species in the host cell cytoplasm, apolipoprotein B mRNA-editing, enzyme-catalytic, polypeptide-like 3G (APOBEC3G) induces hypermutations in the viral genome generating non-functional unstable genomes and SAM domain and HD domain 1 (SAMHD1) deprives the viral reverse transcriptase of the deoxynucleoside building blocks required for its action (Malim and Bieniasz, 2012 and this review series).

Escape from innate immune sensing is therefore paramount to the establishment of productive viral infections. In recent years, several lines of evidence have shown that HIV has evolved highly specialized mechanisms to elude cellular blocks. For example, blocks imposed by restriction factors are mostly overcome through the use of viral accessory proteins (Vpx, Vpr, Nef, Vif, Vpu). Accessory proteins, initially qualified as such because unrequired for *in vitro* replication in permissive cells, are encoded by lentiviral genomes in addition to the essential structural and enzymatic proteins required for mature viral particles production (Gag, Pol, Env, Tat, and Rev). Additional mechanisms deployed by viruses to avoid innate immune sensing include a direct action on the IFN signaling cascade: inhibition of IFN synthesis, IFN receptor decoy and inhibition of IFN signaling (for review Stetson and Medzhitov, 2006; Broz and Monack, 2013). HIV also takes advantage of pre-existing cellular processes. Importantly, while cellular nucleic acid sensors recognize virus-derived nucleic acids and thereby detect incoming virions, they also play crucial roles in cellular metabolism and are usually constitutively expressed. They may therefore detect the presence of nucleic acid species resulting from DNA damage-associated repair mechanism or endogenous retroelement life cycle. Thus, cellular processes co-exist to prevent accumulation of abnormal self-nucleic acids, thereby preventing auto-initiation of pro-inflammatory responses (for review Yan and Hasan). These include major cellular exonucleases that have also been shown to positively impact of HIV life cycle: ribonuclease H2 (RNaseH2) and three prime repair exonuclease 1 (TREX1) (Yan et al., 2010; Genovesio et al., 2011). Importantly, these proteins involved in nucleic acid metabolism belong to a family of genes that, when mutated, lead to the Aicardi-Gouttière syndrome (AGS). This rare autosomal recessive genetic encephalopathy is characterized by neurological dysfunctions, intracranial calcifications, brain atrophy, psychomotor retardation and increased plasma levels of IFN that lead to chronic inflammation (Lebon et al., 1988). We recently established that the SLX4 structure-specific endonuclease regulator complex also acts as a facilitator of HIV infection (Laguette et al., 2014). This finding bears substantial similarities with what was shown for the TREX1 exonuclease. Indeed, similar to TREX1, the SLX4 complex is involved in nucleic acid metabolism and plays crucial roles in the repair of DNA lesions. In addition, the core component of this complex is the SLX4 molecular scaffold that assembles structure-specific endonuclease modules. Biallelic mutations in *SLX4* are involved in the onset of Fanconi anemia (FA), a cancer predisposition syndrome characterized by congenital malformations, hypersensitivity to DNA interstrand cross-linking agents and progressive bone marrow failure (Sasaki and Tonomura, 1973). In addition, FA patients experience heightened pro-inflammatory cytokines levels (Whitney et al., 1996; Rathbun et al., 1997; Dufour et al., 2003; Briot et al., 2008). The latter is a feature shared with AGS patients and supports a potential link between proteins involved in DNA damage response and the development of inflammatory responses. These recent findings also shed a new light on the implication on proteins involved in the maintenance of genomic stability and the HIV life cycle.

## CROSS-TALK BETWEEN CELL CYCLE REGULATION MACHINERY AND VIRAL INFECTIONS

Viruses have been long known to keep a privileged relationship with cell cycle regulatory mechanisms. Indeed, an estimated 20% of human cancers arise from infection with DNA or RNA viruses. Malignancy frequently results from side-tracking of cell cycle regulatory elements. A stricking example is virus-driven oncogenesis that results from subversion of the boundaries between the DNA replication step (S), segregation of sister chromatids (mitosis) and gap phases (G1 and G2). This is frequently achieved through viral non-structural proteins that modulate cell cycle regulators. Transforming viruses essentially subvert the G1/S boundary, thereby pushing cells into proliferation. In the case of the retrovirus HTLV-I, that encodes several potential oncogenes, the well-studied Tax protein is necessary and sufficient to initiate cellular transformation, while the HBz protein is required for its maintenance (Gatza et al., 2003). Similarly, HBx from the DNA virus HBV has the ability to transform immortalized cell lines and to provoke liver cancer in mice (Kim et al., 1991). Small DNA tumor viruses often encode potent oncoproteins that can cause cellular transformation *in vitro* [for example E6/E7 from HPV – for review (Howley and Livingston, 2009)]. In contrast certain viruses such as EBV require the concerted action of several proteins to achieve cellular transformation (Kutok and Wang, 2006).

Importantly, manipulation of the cell cycle or of cell cycle regulators is not solely confined to transforming viruses. Indeed, several DNA and RNA viruses are able to cause cell cycle arrest at the G2/M transition, including HIV (Davy and Doorbar, 2007). The molecular mechanisms underlying virus-induced G2/M arrest vary widely and have been extensively studied. Nonetheless, the understanding of the biological end-point of G2/M arrest remains poor despite suggestions that G2/M arrest may decrease propensity to secrete IFN (Lee and Rozee, 1970), increase RNA production rates (Lee and Rozee, 1970), and overall boost early step of the HIV life cycle (Groschel and Bushman, 2005). Subversion of the host cell cycle by HIV-1 relies on the highly conserved viral protein regulatory (Vpr) protein that causes a potent G2/M arrest in most cycling eukaryotic cells (Di Marzio et al., 1995; He et al., 1995; Jowett et al., 1995; Re et al., 1995; Rogel et al., 1995).

From a mechanistic stand point, Vpr-induced G2/M arrest is a well-documented phenotype. To understand how G2/M arrest is achieved, it is necessary to recapitulate the mechanism underlying this cell cycle check-point (for more details see Guenzel et al, this issue). Indeed, in healthy cells, the G2/M transition is controlled by Cyclin-dependent kinase1:CyclinB1 (CDK1:CCNB1). As cells progress through G2, CDK1:CCNB1 is progressively activated and once the G2/M boundary crossed, the complex is inactivated. The G2/M check-point serves as a quality-control step during the replication of the cellular genome that ensures the transmission to daughter cells of a complete unaltered set of chromosomes. Thus, when genotoxic stress is incurred, entry into mitosis is prevented to provide an opportunity to repair genomic lesions (Stark and Taylor, 2006). This is achieved through preventing CDK1:CCNB1 activation (Sanchez et al., 1997). This response is regulated through a signaling cascade that involves detection of the DNA lesion by the key DNA damage response regulators ataxia-telangiectasia-mutated kinase (ATM), ATM and Rad3-related kinase (ATR), and DNA-dependent protein kinase [DNA-PK; for review (Smith et al., 2010; Sirbu and Cortez, 2013)]. When damaged DNA or unreplicated regions of the genome are detected, these kinases activate downstream CHK1 or CHK2 that in turn inactivate CDK1:CCNB1 (Lopez-Girona et al., 1999).

Similar to what is observed following genotoxic stress, Vpr expression activates ATR, ATM, and the downstream CHK1/CHK2 kinases (Roshal et al., 2003; Munoz et al., 2009), thereby inactivating CDK1:CCNB1. In agreement, treatment of Vpr-expressing cells with caffeine, which inhibits ATR and ATM, relieves the cell cycle block (Poon et al., 1997; Shostak et al., 1999). Intriguingly, although mobilization onto sub-regions of the chromatin of breast cancer susceptibility protein 1 (BRCA1) and γH2ax have been reported upon Vpr expression, it remains unclear whether actual DNA breaks occur in the presence of Vpr and whether these lesions would be the trigger for cell cycle arrest. Rather, the prevailing view is that Vpr mediates ATR-dependent replication stress. Importantly, since the only consensual cellular partner of Vpr for the induction of G2/M arrest is the VPRBP-DDB1-CUL4 E3-ligase complex (Belzile et al., 2007; DeHart et al., 2007; Hrecka et al., 2007; Le Rouzic et al., 2007; Tan et al., 2007; Wen et al., 2007), it was assumed that Vpr would provoke the proteasomal degradation of a cell cycle regulatory element governing the G2/M transition. We recently identified the SLX4 complex as being the Vpr partner required for G2/M arrest (Laguette et al., 2014). Indeed, the structure specific endonucleases ERCC4^XPF^-ERCC1 and MUS81-EME1 together with the SLX4^FANCP^ scaffold protein co-purified with Vpr as well as the poorly characterized TSPYL1 and C20orf94^SLX4IP^ subunits. Vpr binds to the C-terminus of SLX4, inducing the recruitment of VPRBP and kinase-active PLK1. This leads to VPRBP-induced ubiquitination of MUS81 and hyperphosphorylation of EME1, the consequence of which being activation of SLX4-associated MUS81-EME1. Vpr-induced untimely activation of SLX4-bound MUS81-EME1 results in replication stress, ultimately leading to G2/M cell cycle arrest (Laguette et al., 2014).

## VIRAL PROTEIN REGULATORY AND SLX4 COMPLEX REGULATION

### Vpr AND THE FANCONI ANEMIA PATHWAY

SLX4, also as known as FANCP, together with the fifteen additional identified FA or FA-like proteins, is involved in the FA DNA repair pathway. This pathway has been extensively described in reviews (Garner and Smogorzewska, 2011; Su and Huang, 2011; Constantinou, 2012). Briefly, FANCM binds chromatin at damage sites and recruits the E3 ubiquitin ligase activity-containing FA core complex (Kim et al., 2008). The FA core complex monoubiquitinates FANCD2–FANCI and stabilizes them at sites of damage (Garcia-Higuera et al., 2001). FANCD2–FANCI subsequently activate DNA repair proteins, including the SLX4 complex (Yamamoto et al., 2011). The latter is involved in the repair of double strand breaks, interstrand cross-links (ICL), and collapsed/damaged replication forks by homologous recombination (HR; Munoz et al., 2009; Svendsen et al., 2009; Kim et al., 2011; Stoepker et al., 2011). HR allows accurate repair by using the sister chromatid as a template and leads to the formation of four-way DNA structures, Holliday junctions (HJ), that must be removed prior to chromosome segregation. It is important to note that in somatic cells, the favored pathway to remove HJ relies on non-endonucleolytic dissolution by Bloom (BLM)-related helicases, a process that prevents sister chromatid exchanges (Wu and Hickson, 2003). Sister chromatid exchanges are particularly disfavored in somatic cells because they may engender loss of heterozygosity thereby predisposing cells to cancer (Matos et al., 2011, 2013; Gallo-Fernandez et al., 2012; Dehe et al., 2013; Saugar et al., 2013; Szakal and Branzei, 2013). However, in certain cases, for example in the absence of BLM or when the levels of damage incurred are above those that can be salvaged through dissolution, structure-specific endonucleases activities associated with the SLX4 complex may be mobilized (Schwartz and Heyer, 2011; Garner et al., 2013). *In vitro* studies have shown that SLX4–SLX1 (Fekairi et al., 2009; Munoz et al., 2009; Svendsen et al., 2009) and MUS81-EME1 have 5′ and 3′ endonuclease activities, respectively (Boddy et al., 2001; Doe et al., 2002; Ciccia et al., 2003; Gaillard et al., 2003). However, SLX4-associated resolvase activity requires interaction of SLX1 and MUS81-EME1 with the SLX4 scaffold (Castor et al., 2013; Garner et al., 2013; Wyatt et al., 2013).

Because activation of MUS81-EME1 during S phase may cause pathological processing of healthy replication forks (Dehe et al., 2013; Matos et al., 2013; Saugar et al., 2013) and replication stress (Blais et al., 2004; Matos et al., 2013; Szakal and Branzei, 2013), under physiological conditions and when DNA damage is incurred, acquisition of MUS81-EME1 endonuclease activity is under tight regulatory circuits. Those ensure that MUS81-EME1 activity is mostly confined to late G2-early M, when bulk DNA synthesis has been completed (Dehe et al., 2013; Saugar et al., 2013). The molecular mechanism underlying Mus81-Mms4^EME1^ regulation has been extensively studied, and is achieved through phosphorylation of EME^Mms4^ by PLK1^Cdc5^ in budding yeast and in mammalian cells (Matos et al., 2011; Gallo-Fernandez et al., 2012; Saugar et al., 2013) or Cdc2^CDK1^ in fission yeast (Dehe et al., 2013). Importantly, work in mammalian cells has recently shown that MUS81-EME1 is regulated through phosphorylation of EME1 within the SLX4 complex (Castor et al., 2013; Garner et al., 2013; Wyatt et al., 2013).

**FIGURE 1:**
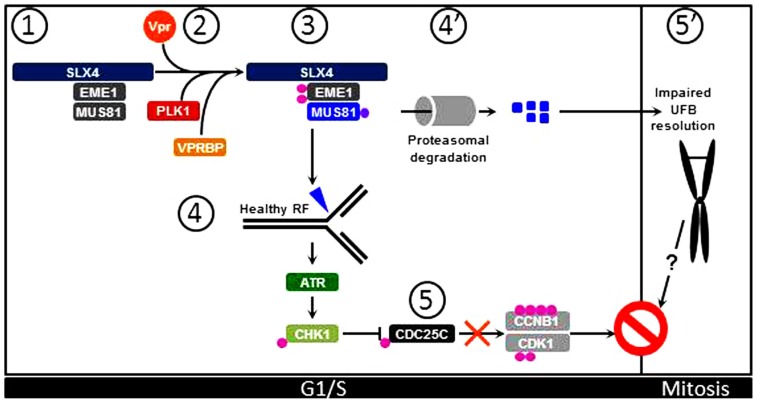
**Vpr induces G2/M arrest through activation of the SLX4 complex.** (1) Under physiological conditions, inactive MUS81-EME1 interact with the SLX4 scaffold. (2) Upon Vpr expression, PLK1 and VPRBP are recruited to SLX4. (3) PLK1 phosphorylates EME1 while VPRBP causes ubiquitination of MUS81. (4) These posttranslational modifications contribute to activation of SLX4-bound MUS81-EME1 that can process healthy replication forks (RF) in cycling cells. (5) This leads to activation of the ATR signaling pathway and subsequent activation of CHK1. Activated CHK1 provokes inhibitory phosphorylation of CDC25C, leading to inhibition of CCNB1:CDK1 and cell cycle arrest at the G2/M transition. (4′) In addition, ubiquitinated MUS81 molecules are degraded by the proteasome machinery, leading to decreased steady-state levels of MUS81. (5′) Consequently, UFBs are not processed and persist in Mitosis. This possibly contributes to overall G2/M arrest in Vpr expressing cells.

Importantly, interaction of Vpr with the SLX4 scaffold protein induces recruitment of VPRBP and kinase-active PLK1, thereby activating the MUS81-EME1 endonuclease module independently of the cell cycle stage (**Figure 1**). This results in replication stress as visualized by accumulation of FANCD2 on sub-regions of the chromatin that likely mark the sites of abnormal processing of replication intermediates (Naim and Rosselli, 2009). Thus, as supported by previous work, Vpr causes cell cycle arrest through a S phase-dependent mechanism (Li et al., 2010), which is congruent with activation of the ATR pathway in Vpr expressing cells (Roshal et al., 2003; Lobrich and Jeggo, 2007). Interestingly, SLX4 has also been identified as an ATR substrate (Matsuoka et al., 2007; Mu et al., 2007) and in yeast, phosphorylation of Eme1 requires Rad53^ATR^ activation (Dehe et al., 2013). Thus, aberrant processing of stalled replication forks by Vpr-activated SLX4-associated MUS81-EME1 would cause replication stress, ATR-CHK1 pathway activation, resulting in inhibition of CDC25C. This signaling cascade will ultimately lead to inability of CDC25C to activate CCNB1:CDK1 and thus result in G2/M arrest (**Figure 1**).

### Vpr AND GENOMIC INSTABILITY

The MUS81-EME1 endonuclease module plays an important role in the removal of ultrafine DNA bridges (UFBs). These DNA structures that arise from regions of the genome that replicate at slower rates, such as centromeres and common fragile sited (CFS), are formed during the S phase and can be visualized after chromosome condensation in mitotic cells. They form bridges between sister chromatids that must be removed prior to chromosome segregation. Absence of MUS81-EME1 results in non-processing of UFBs and thus leads to CFS-associated chromosomal instability and mitotic catastrophe (Chan et al., 2009; Chan and Hickson, 2011; Wechsler et al., 2011; Naim et al., 2013; Ying et al., 2013). Accumulation of UFBs therefore causes cell cycle arrest at the G1/S transition. Intriguingly, Vpr targets MUS81 for ubiquitination by VPRBP, leading to decreased levels of MUS81 prior to G2/M arrest (Laguette et al., 2014). Since a stark increase of FANCD2 twin foci that mark the edges of UFBs (Chan et al., 2009) occurs in the presence of Vpr, this indicates that, although not complete, decreased MUS81 levels in Vpr-expressing cells may be sufficient to impair UFBs untangling prior to mitosis (Chan et al., 2009; Wechsler et al., 2011; Naim et al., 2013; Ying et al., 2013). However, Vpr-associated replication stress prevents completion of G2. This likely prevents the occurrence of mitotic catastrophe. While it is possible that additional Vpr-associated functions may prevent cells from exiting mitosis, one may also speculate that steric hindrance imposed by UFBs tying together sister chromatids may also contribute to the extent of G2/M arrest witnessed in Vpr-expressing cells (**Figure 1**). Thus, the complete sequence of events leading from SLX4 complex premature activation to cell cycle arrest by Vpr requires further investigations.

## VIRAL PROTEIN REGULATORY AND INNATE IMMUNITY

One interesting feature of Vpr is that disruption of the corresponding open reading frame (ORF) results in inefficient viral spread *ex vivo* particularly in primary macrophages (Connor et al., 1995) while its most studied molecular function is to halt cell cycle progression. This conundrum has puzzled the HIV field for several years but little was understood about how these two observations can be reconciled until recent work (Laguette et al., 2014).

**FIGURE 2:**
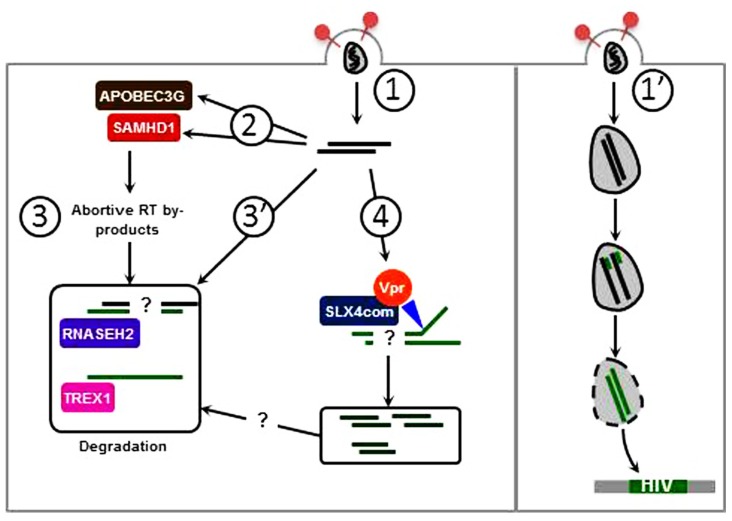
**Vpr-induced SLX4 complex activation promotes escape from innate immune sensing.** (1) Premature delivery of viral genomes in the cytoplasm of host cells may lead to recognition by nucleic acid sensors. (2) Editing by APOBEC3G and dNTP hydrolysis by SAMHD1 induce viral DNA instability and impair reverse transcription. Of note, a nuclease activity has been described for SAMHD1 which may target viral nucleic acids. (3) APOBEC3G and SAMHD1 contribute to generate abortive RT by-products which are taken care of by cellular exonucleases: RNaseH2 degrades DNA:RNA hybrids while TREX1 degrades ssDNA preferentially. (3′) Abortive nucleic acid intermediates may also be directly degraded by RNaseH2 and TREX1. (4) The SLX4 structure specific endonuclease complex is activated by Vpr and likely cleaves dsDNA. Whether these dsDNA fragments are further degraded by cellular exonucleases or may be recognized by nucleic acid sensors remains questioned. (1′) When uncoating is correctly orchestrated, viral nucleic acids are protected from mechanisms described in 1–4, and ensures delivery of viral genomes in the nucleus.

Infection with an HIV-1 molecular clone harboring a deletion of the Vpr ORF causes an increase of IFN production as compared to infection with wild type HIV-1 (Okumura et al., 2008; Doehle et al., 2009; Laguette et al., 2014). This HIV-induced IFN production is augmented following SLX4 complex subunits (SLX4, VPRBP, and MUS81) knock-down, suggesting that the presence of the SLX4 complex is required for inhibition of HIV-dependent type 1 IFN production. Furthermore, the SLX4 complex binds HIV-1-derived reverse transcribed DNA in presence of Vpr, suggesting that Vpr is required for this interaction. In addition, in the absence of SLX4, there is an increase of HIV DNA in infected cells. This further suggests that the SLX4 complex is required to degrade excess HIV-derived nucleic acids susceptible of triggering innate immune responses. While the MUS81-EME1 endonuclease module appears to be required for this process one cannot exclude contribution of additional SLX4-bound endonucleases. Indeed, SLX4–SLX1 interaction is required for SLX4 complex associated resolvase activity and SLX1 expression is required for Vpr-mediated cell cycle arrest (Laguette et al., 2014). Overall, similar to TREX1 and RNaseH2, the SLX4 complex would prevent sensing of excess nucleic acids derived from HIV reverse transcription (**Figure 2**). Importantly, RNaseH2 and TREX1 preferentially degrade DNA within DNA:RNA hybrids (Haruki et al., 2002) and ssDNA substrates, respectively (Mazur and Perrino, 2001) while SLX4-bound MUS81-EME1 presumably target dsDNA structures (Fadden et al., 2013). While all these nucleic acid species arise in the course of HIV reverse transcription, the relative contribution of these nucleases remains to be evaluated. Furthermore, SLX4-mediated nucleic acids processing would lead to the generation of DNA fragments that likely require further processing to avoid recognition. This leaves several questions open amongst which are: what is the sensor triggered in the absence of SLX4 and what are the nucleases mobilized to clear SLX4-processed DNA fragments? These processes may rely on previously identified key players in viral life cycles. However, one must bear in mind that the SLX4 scaffold can bind to additional proteins involved in DNA metabolism and is as such involved in several additional pathways, including Telomere maintenance and DNA mismatch repair (Svendsen et al., 2009). Whether these may intervene in the degradation of virus derived nucleic acids is also to be explored.

As previously mentioned, FA is associated with high production of pro-inflammatory cytokine in patients. This can be recapitulated *in vitro* in *SLX4*-deficient patient cells (Kim et al., 2011) and also in mouse embryonic fibroblasts knocked-out of *MUS81* (McPherson et al., 2004) through the activation of NF-κB pathway (Laguette et al., 2014). This leads to the establishment of an antiviral state that likely accounts for the inability of these cells to support efficient HIV replication. While this bears similarities with what is observed in TREX1 deficiency, the endogenous trigger for SLX4 complex activation remains unknown. Those may include, nucleic acids derived from processing of aberrant replication intermediates or endogenous retroviruses. Elucidating the trigger for spontaneous upregulation of pro-inflammatory cytokines in FA is likely to be the next horizon in the field. This would possibly provide insight into the molecular basis of FA-associated chronic inflammation. In addition, Vpr has been shown to modulate immune responses at additional levels. This includes impairment of DC/macrophage maturation, disruption of natural killer T cells effector functions, increased apoptosis of cytotoxic T cells and disruption of T cell activation pathways (reviewed in Ayinde et al., 2010). Thus, Vpr compromises the establishment of adaptive immune responses. How inhibition of pro-inflammatory cytokines by Vpr through activation of SLX4 complex contributes to this process remains to be explored.

## DNA DAMAGE RESPONSE AND INNATE IMMUNITY

Initiation of the DNA damage response usually requires recognition of abnormal nucleic acid species in the nucleus and the triggering of a signaling cascade that orchestrates repair. This process bears similarities with what is witnessed when virus-derived nucleic acids are delivered into host cells. Those are recognized by sensors that trigger a signaling cascade aiming at clearing the infection. It has been widely speculated that the nucleic acid-based repository of the information required for *de novo* virus production is difficultly modified by viruses to promote escape; it constitutes a prime target for cellular sensors. Recent work has placed key players of the DNA damage response on the front line of pathogen recognition. For example DNA-PK has been shown to act as a PRR for DNA and RNA viruses (Zhang et al., 2011; Ferguson et al., 2012). Indeed, DNA-PK is involved in DNA damage response, more particularly in the repair of double-strand breaks and these functions are related to its nuclear localization (for review Davis and Chen, 2013). However, this complex is also found in the cytoplasm where it can bind nucleic acids and activates the production of IFN. This highlights how overlapping mechanisms have evolved for the recognition of pathological nucleic acid species. Furthermore, inflammation impacts every step of tumorigenesis, from initiation to metastatic progression. Tumor-promoting inflammation may either result from environmental factors, as clearly identified in the case of exposure to asbestos for example, or from chronic viral infections and attempts of the immune system to eliminate those. This results in a feed-forward regulatory loop that favors chronic production of pro-inflammatory cytokines, supporting tumorigenesis. Indeed, it is recognized that subclinical, often undetectable, inflammation increases cancer risk (reviewed in Grivennikov et al., 2010). Thus, persistent DNA damage or inability to repair broken DNA may lead to tumor-promoting chronic inflammation (Zheng et al., 2007; Rodier et al., 2009).

Cellular mechanisms exist that serve to avoid the accumulation of pathological nucleic acid species susceptible of triggering innate immune responses. These include the previously mentioned TREX1 exonuclease and the SLX4 complex. Intriguingly, TREX1 that was initially described to be involved in DNA base excision repair (Mazur and Perrino, 2001), also degrades ssDNA derived from aberrant replication intermediates and thus similar to the SLX4 complex is involved in DNA damage response (Yang et al., 2007; Gehrke et al., 2013). Thus, like in TREX1 deficiency, absence of the SLX4 complex may lead to accumulation of pathological nucleic acids in the cytoplasm. Recognition of those by a yet to be identified sensor, activates the immune system.

Importantly, HIV is not the sole virus affected by cellular enzymes involved in DNA metabolism. Indeed, several DNA viruses can be targeted by cellular factors involved in DNA damage response. For example, the genome of the Adenovirus or Herpes Simplex Virus type 1 (HSV-1) can be targeted by protein complexes that control the non-homologous end-joining DNA repair pathway [reviewed in Weitzman et al. (2010)]. These viruses have evolved potent ways of counteracting these proteins that operate as potential restriction factors. This can be compared to what is witnessed during HIV infection in the presence of SAMHD1. Indeed, this HIV restriction factor has been shown, in addition to depleting the dNTP pool (Lahouassa et al., 2012), to have *in vitro* nuclease activity (Beloglazova et al., 2013).

While there is accumulating evidence that proteins involved in DNA repair are involved in viral life cycles, recent work has also shown that proteins previously identified as counteractors of HIV infection are in fact involved in the DNA damage response. This is the case for APOBEC proteins where mutation patterns were found in human cancers (Leonard et al., 2013; Roberts et al., 2013) and SAMHD1. In the case of SAMHD1, recent work has highlighted that SAMHD1 may qualify as a tumor suppressor gene, and thus play roles in DNA damage response, through its ability to regulate the dNTP pool (Clifford et al., 2014; Kretschmer et al., 2014), the levels of these being important for genome stability (Mathews, 2006). Although this SAMHD1 function has been essentially described to be involved in HIV restriction, it may also be related to increased IFN production in AGS. Moreover, RNaseH2, as previously mentioned, is also involved in AGS and degrades RNA in DNA:RNA hybrids and thus may also prevent chronic inflammation (Rice et al., 2007). Importantly, both SAMHD1 and APOBEC3G have been shown to control endogenous retrotransposition (Esnault et al., 2006; Zhao et al., 2013). Since absence of SAMHD1 also correlates with increased pro-inflammatory cytokine production, one may also speculate about the trigger for this response and whether there is a correlation between inefficient DNA repair or endogenous retroelement retrotransposition in the absence of SAMHD1 and chronic inflammation. Similarly, the origin of chronic inflammation in *SLX4*-deficiency remains to be identified and may include residual nucleic acids resulting from processing of aberrant replication intermediates or endogenous retroelements.

## CONCLUSION

Although host cells oppose numerous blocks to HIV replication, several mechanisms have evolved to counteract those. While defective viruses may elicit pro-inflammatory responses, viruses that establish productive infections remain mostly protected from cellular defenses. Viral accessory proteins are specialized in mediating this escape, in part through counteraction of cellular restriction factors. Interestingly, restriction factors have also been recently reported to play a role in detection of viral infections (PRR). Thus, viral accessory proteins, though their degradation simultaneously achieve escape from recognition and overthrowing of mediators of the antiviral responses. This complex array of interactions between innate immune responses and viral replication is still poorly understood. While new insight into the role of the DNA damage response machinery in this process may add a further layer of complexity, this may also provide with an additional way to identify sensors that detect incoming viruses and escape mechanisms.

In addition to the described role of Vpr in arresting the cell cycle and promoting escape from innate immune sensing, this HIV accessory protein has been shown to play several additional roles in HIV life cycle. Indeed, Vpr contributes to fidelity of reverse transcription (Stark and Hay, 1998), nuclear transport of the pre-integration complex (Heinzinger et al., 1994). Vpr also promotes the transactivation of LTR promoter (Felzien et al., 1998), and induction of apoptosis (Stewart et al., 1997). Do these also result from Vpr-induced activation of the SLX4 complex or do they rely on additional interactions established by Vpr? Importantly, in addition to interacting with structure-specific endonucleases, the SLX4 molecular toolkit also recruits MSH2–MSH3 and TRF2–RAP1 (Svendsen et al., 2009) and is involved in additional cellular functions, including Telomere maintenance, which may be altered upon binding to Vpr. For example, since SLX4 inhibits over-lengthening of telomeric ends, Vpr-induced activation of the SLX4 complex may lead to Telomere shortening and cell death. Whether this is related to increased apoptosis witnessed in Vpr-expressing cells is yet to be explored.

The discovery of the SLX4 complex as being involved in inhibition of pro-inflammatory responses opens new avenues in the understanding of the interplay between innate immune responses and HIV infection. This work also opens new perspectives in the understanding of the molecular mechanism underlying cancer related chronic inflammation. Based on the fact that pro-inflammatory cytokine production is witnessed in all cancers, one may anticipate that additional DNA damage repair mechanisms may be involved in pathogen recognition and inhibition of spontaneous pro-inflammatory cytokine production.

## Conflict of Interest Statement

The authors declare that the research was conducted in the absence of any commercial or financial relationships that could be construed as a potential conflict of interest.
